# Curcumin and Cancer-Related Inflammation

**DOI:** 10.3390/nu18101636

**Published:** 2026-05-21

**Authors:** Kaitlyn LeBlanc, Emilee Brewer, Sita Aggarwal

**Affiliations:** Cancer Prevention Laboratory, Southeastern Louisiana University, Hammond, LA 70403, USA; leblanckait@gmail.com (K.L.); emilee.brewer92@gmail.com (E.B.)

**Keywords:** inflammation, reactive oxygen species, cytokines, nuclear transcription factor–kappa B, signal transducer and activator of transcription 3, curcumin

## Abstract

Chronic inflammation is a well-established risk factor for cancer progression. This review aims to determine how persistent inflammatory signaling reshapes the tissue microenvironment to favor tumor cell proliferation, survival, and progression. It also discusses the role of cytokines such as IL-6 and TGF-β, reactive oxygen species (ROS), and the transcription factors NF-κB and STAT3 in inflammation and in the tumor microenvironment. Sustained activation of these pathways promotes genomic instability, loss of tumor suppressor gene function, enhanced oncogene expression, and resistance to apoptosis, collectively facilitating malignant transformation and tumor development. The key novelty of this review lies in integrating these interconnected networks with new evidence to clarify how they drive cancer initiation and progression. Furthermore, we discuss the therapeutic potential of plant-derived bioactive compounds, with a particular emphasis on curcumin. Curcumin exhibits significant anti-inflammatory and anticancer effects through inhibition of NF-κB and STAT3 signaling and its downstream targets, thereby attenuating inflammation-driven tumorigenesis. However, its clinical application is limited by poor bioavailability. Finally, this review highlights current strategies to overcome these limitations and future directions for optimizing curcumin-based interventions in inflammation-associated diseases.

## 1. Introduction

Chronic inflammation and oxidative stress are central drivers of tumor initiation, progression, metastasis, and therapeutic resistance. Chronic inflammation alters the tumor microenvironment in ways that promote malignant transformation. Studying the relationship between chronic inflammation and tumorogenesis helps explain how normal physiological processes become dysregulated and contribute to cancer initiation and progression. Pro-inflammatory cytokines such as interleukin-6 (IL-6), tumor necrosis factor-α (TNF-α), and transforming growth factor-β (TGF-β) play pivotal roles in shaping the tumor microenvironment (TME), promoting immune evasion, enhancing survival signaling, and facilitating resistance to chemotherapy and radiotherapy [1,2,3]. These cytokines regulate multiple hallmarks of cancer, including proliferation, angiogenesis, metastasis, metabolic reprogramming, and protection against therapy-induced DNA damage.

Persistent activation of the transcription factors nuclear factor kappa B (NF-κB) and signal transducer and activator of transcription 3 (STAT3), together with their downstream inflammation-dependent enzymes cyclooxygenase (COX-2) and inducible nitric oxide synthase (iNOS), further amplifies tumor-promoting signaling and contributes to inflammation-associated malignancies [1,2,3]. Reactive oxygen species (ROS), generated during chronic inflammation, also contribute(s) to genomic instability and activate redox-sensitive pathways that support tumor progression and metastasis [4,5].

However, IL-6, TNF-α, TGF-β and ROS exhibit pleotropic roles: physiological concentrations are required for normal cell signaling and homeostasis, whereas dysregulated production promotes cancer cell survival, proliferation, and adaptation to stress, highlighting their therapeutic potential. Targeting inflammatory mediators and redox-regulated pathways has therefore emerged as a promising anticancer strategy.

To reduce the side effects of radiation and chemotherapies, there is increasing interest in identifying safer alternatives, particularly natural compounds that can target multiple pathways involved in inflammation and cancer. Plant-derived compounds have contributed significantly to modern oncology, with agents such as paclitaxel and the vinca alkaloids serving as established chemotherapeutics. Curcumin, a bioactive polyphenol from *Curcuma longa*, demonstrates anti-inflammatory, antioxidant, chemopreventive, and pro-apoptotic properties through inhibition of NF-κB/STAT 3 signaling. Exploring such compounds supports the development of novel, plant-based therapeutic strategies. Curcumin exhibits promising anticancer effects in preclinical studies by inhibiting proliferation, inducing apoptosis, and modulating NF-κB/STAT3 and related pathways [6,7]. Curcumin has shown synergistic effects with gemcitabine in pancreatic cancer models [8], and strategies such as co-administration with piperine may enhance its clinical utility [9]. Although, curcumin exhibits promising anticancer and disease-modulating properties in preclinical studies, its clinical application is presently limited to use as a complementary (adjuvant) therapy alongside standard treatments rather than as a standalone or alternative therapy [10]. Clinical evidence remains limited and its benefits remain largely confined to adjunctive use, partly due to its poor bioavailability [11,12].

Investigating these limitations is important for advancing drug delivery systems and improving therapeutic efficacy. Although substantial research links inflammation and cancer, gaps remain in fully understanding how persistent inflammatory signaling drives genomic instability and therapy resistance. This makes the topic both relevant and timely for further exploration. Collectively, modulation of inflammatory and oxidative pathways—particularly through naturally derived compounds—represents a promising avenue for improving cancer prevention and therapy.

## 2. Role of Cytokines

### 2.1. Interleukin-6

Among the diverse mediators present in the tumor microenvironment, interleukin-6 (IL-6) has emerged as a central regulator linking chronic inflammation to cancer progression and therapeutic resistance [13]. IL-6 is a pleiotropic cytokine that regulates immune responses, hematopoiesis, tissue repair, and metabolic balance. Under physiological conditions, IL-6 production is rapid and transient, supporting host defense during infection or tissue injury. However, persistent IL-6 expression and sustained receptor engagement shift its role from protective to pathogenic, contributing to chronic inflammatory diseases and malignancies [13,14].

In the context of cancer, IL-6 is frequently elevated across multiple tumor types and is often dysregulated, promoting tumorigenesis by orchestrating key hallmarks of cancer, including enhanced cell proliferation, survival, angiogenesis, invasion, metastasis, and metabolic reprogramming [15]. Importantly, IL-6 signaling has been strongly implicated in resistance to chemo- and radiotherapy. By activating downstream pro-survival and antioxidant pathways, facilitating DNA damage repair, and suppressing apoptosis, IL-6 enables tumor cells to withstand therapy-induced oxidative stress and genotoxic insults [15,16].

IL-6 exerts its biological effects through binding to the membrane-bound IL-6 receptor (IL-6Rα) or soluble IL-6R, followed by association with the signal-transducing co-receptor gp130 [14]. This interaction activates several downstream pathways, most prominently the STAT3 pathway, as well as the MAPK/ERK and PI3K/AKT cascades [13]. Canonical IL-6/STAT3 signaling drives transcription of genes involved in cell proliferation and survival (e.g., cyclins and c-Myc) and anti-apoptotic responses (e.g., Bcl-2, Bcl-xL, and survivin), as well as immune evasion and inflammation.

In many tumor types, including breast [17], colorectal [18], pancreatic [19], lung [20] and hepatocellular carcinomas [21], constitutive activation of IL-6/STAT3 signaling establishes a feed-forward inflammatory loop. Tumor cells, cancer-associated fibroblasts (CAFs), tumor-associated macrophages (TAMs), and other stromal components secrete IL-6, reinforcing paracrine and autocrine signaling that sustains malignant behavior [22]. Collectively, these findings underscore that dysregulated IL-6 signaling not only sustains tumor growth and progression but also establishes a protective microenvironment that fosters therapeutic resistance. Thus, targeting the IL-6/IL-6R/JAK/STAT3 axis—through monoclonal antibodies (e.g., IL-6 or IL-6R blockers), JAK inhibitors, or STAT3 inhibitors—is an active area of translational research aimed at improving outcomes in patients with IL-6-driven malignancies [22]. Overall, these lines of evidence clearly show that targeting IL-6 could be a promising therapeutic strategy in cancer.

### 2.2. Transforming Growth Factor-β

The functions of transforming growth factor-β (TGF-β) are highly dependent on the cell type and cellular context. Evidence suggests that TGF-β signaling exerts a biphasic, “double-edged sword” effect during tumor development [23]. In normal tissues and early-stage tumor cells, activation of the TGF-β pathway primarily functions as a tumor suppressor by inducing cell-cycle arrest, promoting apoptosis, and maintaining tissue homeostasis [24]. However, as tumors progress, cancer cells often acquire mechanisms that attenuate the growth-inhibitory effects of TGF-β while retaining or enhancing its pro-tumorigenic activities. In advanced-stage cancers, TGF-β signaling can therefore shift toward an oncogenic role, facilitating epithelial–mesenchymal transition (EMT), invasion, metastasis, immune evasion, and resistance to chemotherapy and targeted therapies [24,25,26].

TGF-β plays a pivotal role in shaping the immunosuppressive TME in oral squamous cell carcinoma (OSCC). It promotes immune evasion by inducing the differentiation and expansion of regulatory T cells (Tregs) and facilitating the activation of cancer-associated fibroblasts (CAFs), both of which contribute to tumor progression. Concurrently, TGF-β suppresses anti-tumor immunity by inhibiting the cytotoxic activity and effector functions of cytotoxic T lymphocytes (CTLs) and natural killer (NK) cells. Through these coordinated mechanisms, TGF-β fosters an immune-tolerant microenvironment that supports OSCC growth and progression [27].

TGF-β-induced epithelial–mesenchymal transition (EMT) is a critical driver of tumor metastasis [28]. It has been shown that inhibition of TGF-β activity inhibited HNF1A-AS1/oncostatin M-mediated EMT in gastroenteropancreatic neuroendocrine neoplasms [29]. Moreover, studies indicate that pancreatic cancer cells themselves do not secrete TGF-β; rather, TGF-β secretion at the tumor site results from infiltrating granulocytes (predominantly neutrophils), leading to overproduction of collagens [30]. Taking together, these findings clearly show that targeting TGF-β signaling could be a promising therapeutic approach in cancer.

### 2.3. Tumor Necrosis Factor Alpha

Tumor necrosis factor (TNF)-α is a pleiotropic cytokine with key roles in inflammation [31]. It promotes inflammatory responses in immune cells, including T and B lymphocytes, as well as in non-immune, tissue-resident cells such as fibroblasts and epithelial cells [32]. TNF-α is mainly produced by activated macrophages and monocytes, and its expression is rapidly induced in response to pathogen-associated molecular patterns (PAMPs) via toll-like receptors (TLRs), elevated levels of other cytokines (e.g., IL-1 and IFN-γ), and various forms of cellular stress or tissue injury. These features make TNF-α one of the earliest cytokines released during inflammation [33,34].

TNF-α is a type II transmembrane protein that functions either as a membrane-bound signaling molecule or as a soluble cytokine generated through proteolytic cleavage. Binding of TNF family ligands to their receptors triggers the recruitment of intracellular adaptor proteins and activates multiple downstream signaling pathways [35]. Based on differences in their intracellular domains, members of the tumor necrosis factor receptor (TNFR) superfamily are broadly classified into three major groups. Although TNF receptor-associated factor (TRAF) proteins lack intrinsic enzymatic activity, they function as adaptor molecules that initiate diverse signaling cascades regulating proliferation, differentiation, and cell death [35,36].

TNF-α was originally identified as a factor capable of suppressing tumor cell proliferation and was linked to tumor necrosis and a variety of inflammatory diseases [37,38]. Apoptosis induced through TNF superfamily receptors involves adaptor proteins such as FAS-associated death domain (FADD) and TNFR-associated death domain (TRADD) [35]. However, accumulating evidence now recognizes that overexpression of TNF-α also confers potent pro-tumorigenic properties [39,40,41]. TNF-α promotes angiogenesis by recruiting endothelial progenitor cells at the sites of tissue damage during inflammatory signaling [42], and its overexpression induces chemoresistance in leukemia, pancreatic ductal adenocarcinoma, and breast cancer cells [39,40,41].

Cancer cell survival signaling activates the NF-κB signaling cascade and other pathways downstream of TNF-α, depending on the cellular context [35]. Activation of NF-κB by TNF-α and other TNFR superfamily members is primarily mediated through the recruitment of distinct TRAF proteins. At the molecular level, TNF-α acts through several mechanisms. First, activation of TNF- receptor-1 (TNFR1) predominantly recruits TRADD, which can then further associate with TRAF2, TRAF1, and receptor-interacting protein (RIP), resulting in NF-κB activation that promotes cell survival and counteracts apoptotic signaling [36]. Second, activation of TRAF-interacting motifs (TIMs) promotes recruitment of TRAF proteins, stimulating multiple signaling pathways, including NF-κB [35]. Third, decoy receptors compete with signaling-component receptors for ligand binding, thereby modulating downstream responses [35].

Consistent with these observations, anti-TNF-α antibodies and other TNF-α antagonists have demonstrated therapeutic efficacy in multiple experimental models of common epithelial cancers [39,40,41,43] and in alleviating inflammation associated with several autoimmune diseases [32]. Collectively, these findings indicate that TNF-α remains an important therapeutic target at the intersection of inflammation and cancer.

## 3. Reactive Oxygen Species

Reactive oxygen species (ROS) are highly reactive oxygen-derived molecules generated as natural byproducts of cellular metabolism and are essential for maintaining physiological processes, including redox signaling and host defense. ROS is a collective term that includes superoxide (O_2_•^−^), hydrogen peroxide (H_2_O_2_), hydroxyl radical (OH•), singlet oxygen (^1^O_2_), peroxyl (LOO•), alkoxyl (LO•), lipid hydroperoxide (LOOH), peroxynitrite (ONOO^−^), hypochlorous acid (HOCl), and ozone (O_3_). Several ROS, such as superoxide, hydroxyl radical, peroxyl, and alkoxyl, contain unpaired electrons and are therefore classified as free radicals, whereas others lacking unpaired electrons—such as hydrogen peroxide, peroxynitrite, hypochlorous acid, and ozone—are considered non-radical species [44].

The thioredoxin system is a major cellular system which maintains intracellular redox balance by preserving high levels of reduced protein thiols [45]. Thioredoxin reductase (TrxR) is one of the enzyme of the thioredoxin system that is overexpressed in tumor cells, provides resistance against oxidative stress, and supports tumor cell proliferation, anti-apoptosis and immune-mediated damage [46]. Accumulating evidence indicates that dysregulated ROS production plays a pivotal role in chronic inflammation and tumor progression [5,47,48]. Within the chronically inflamed tumor microenvironment, persistent activation of inflammatory cells enhances ROS generation, thereby establishing a pro-oxidant milieu [49]. Elevated ROS levels can directly induce mitochondrial genomic instability through oxidative DNA damage [4,48] or indirectly modulate redox-sensitive signaling pathways that regulate proliferation, EMT, invasion, and metastasis. Such mechanisms have been implicated in the progression and dissemination of multiple malignancies, including breast, lung, liver, and colorectal cancers [5].

Importantly, ROS functions as a double-edged sword in cancer biology. Sustained intracellular ROS can act as secondary messengers in signaling cascades that promote and maintain an oncogenic phenotype, supporting tumor initiation and metastasis. At the same time, excessive ROS accumulation can surpass the antioxidant capacity of tumor cells, leading to growth arrest or cell death, thereby functioning as anti-tumorigenic agents [50,51,52]. ROS also contributes to malignant transformation and progression to metastasis [51,53].

Cancer cells undergo metabolic reprogramming to sustain rapid proliferation, which requires increased adenosine triphosphate production, elevated biosynthetic activity, and tight regulation of the intracellular redox balance [51,54]. Consequently, mitochondrial ROS production is elevated, but this increase is counteracted by upregulated antioxidant scavenging systems to maintain redox homeostasis [51,55]. In parallel, inflammatory cytokines such as TNF-α critically regulate ROS and reactive nitrogen species signaling, thereby influencing innate immune cell function under both physiological and inflammatory conditions. TNF-α-mediated redox signaling pathways modulate immune cell activation, survival, and effector responses, further linking chronic inflammation, oxidative stress, and tumor progression [56]. This functional duality has generated strong interest in exploiting ROS modulation as a therapeutic strategy to enhance tumor-selective oxidative damage and improve precision targeting.

## 4. Nuclear Factor Kappa B

TNF-α, IL-6, TGF-β, and ROS activate key inflammatory signaling mediators such as NF-κB and exhibit extensive crosstalk with each other in cancer growth and cancer metastasis [57,58,59,60,61]. Activated NF-κB plays a dual role in inflammation. In innate immune cells, activated NF-κB promotes the production of pro-inflammatory mediators (e.g., neutrophils and macrophages) and pro-inflammatory cytokines (e.g., TNF-α and IL-6) that facilitate pathogen clearance, initiate tissue repair, and maintain innate immunity [62]. In adaptive immunity, activated NF-κB regulates the activation and differentiation of both T and B lymphocytes. It contributes to the development of effector T-cell subsets such as Th1, Th2, Th17, and regulatory T cells (Tregs) in coordination with co-stimulatory signals from molecules such as CD28. In B cells, antigen recognition through the B-cell receptor activates NF-κB, promoting proliferation, differentiation into plasma cells, and antibody production. T-cell-derived co-stimulatory signals, mediated by cytokines such as IL-4 and interactions involving the CD40 ligand, further enhance NF-κB activation in B cells, facilitating class-switch recombination and terminal differentiation [62,63].

However, sustained or aberrant activation of NF-κB can drive the development of chronic inflammatory disorders, autoimmune diseases, and cancer. In such conditions, the negative feedback mechanisms that normally resolve inflammation are overwhelmed by persistent NF-κB signaling. This intricate signaling network underscores the central role of NF-κB in regulating immune responses, inflammatory processes, and tumorigenesis [64]. NF-κB is a ubiquitously expressed transcription factor implicated in numerous pathological conditions, particularly inflammation-associated cancer. It regulates the transcription of several pro-inflammatory cytokines, including TNF-α, thereby promoting the survival of transformed cells through suppression of apoptotic pathways [38,65,66].

In its resting state, NF-κB remains sequestered in the cytoplasm through tight association with specific inhibitory proteins known as IκBs. These inhibitors belong to a gene family that includes IκBα, IκBβ, IκBγ, IκBε, Bcl-3, p100, and p105. Upon stimulation by agents such as TNF-α, IκBα is phosphorylated at serine residues 32 and 36, ubiquitinated at lysine residues 21 and 22, and subsequently degraded via the proteasomal pathway. This degradation unmasks the nuclear localization signals of the p50–p65 heterodimer. The p65 subunit is then phosphorylated, enabling its translocation to the nucleus, where it binds to specific DNA sequences and initiates gene transcription of pro-inflammatory cytokines and chemokines [38,67].

NF-κB influences all six hallmarks of cancer by transcriptionally activating genes involved in cell proliferation, angiogenesis, metastasis, tumor promotion, inflammation, and cell survival [42,68,69,70,71,72,73]. Because tumor cells frequently exploit NF-κB to develop resistance to chemotherapy and radiation, targeting NF-κB activation represents a promising strategy to enhance the effectiveness of conventional anticancer treatments [74,75]. Although NF-κB activity is tightly regulated in most normal cells, it is constitutively activated in several malignancies where it contributes to tumor growth and chemoresistance [68]. Consequently, pharmacologic inhibition of NF-κB is considered a promising therapeutic strategy for cancer treatment.

## 5. Curcumin

Approximately 25% of prescription drugs in the United States are derived from plant sources, many of which were identified through systematic investigation of traditional and folkloric medicine [76]. Notable examples include paclitaxel (Taxol), originally isolated from the Pacific yew tree, and the vinca alkaloids vinblastine and vincristine, derived from the rosy periwinkle (*Catharanthus roseus*). These plant-derived agents have been extensively characterized and remain integral components of modern chemotherapeutic regimens, demonstrating efficacy comparable to or exceeding that of many synthetic drugs [76].

Another widely studied medicinal plant is *Curcuma longa* L. (Zingiberaceae), a perennial herb cultivated throughout tropical regions of Asia. Its rhizome, commonly known as turmeric, is used both as a culinary spice and as a traditional medicinal agent [77]. The bioactive fraction of turmeric extract contains 60–70% curcumin (diferuloyalmethane), 20–27% demethoxycurcumin, and 10–15% bisdemethoxycurcumin [78]. Curcumin (diferuloylmethane), the principal bioactive constituent of turmeric, exhibits a broad spectrum of pharmacological activities, including anti-inflammatory and antioxidant properties [79], as well as chemopreventive and chemotherapeutic potential [80,81]. Owing to its favorable safety profile and low systemic toxicity, curcumin has attracted considerable interest as a candidate anticancer agent. Preclinical studies have demonstrated that curcumin exerts potent antiproliferative and pro-apoptotic effects in vitro [82,83] and suppresses breast [84,85,86,87], colon [88], liver [89,90,91], pancreatic [92], and cervical cancers [93,94].

**Figure 1:**
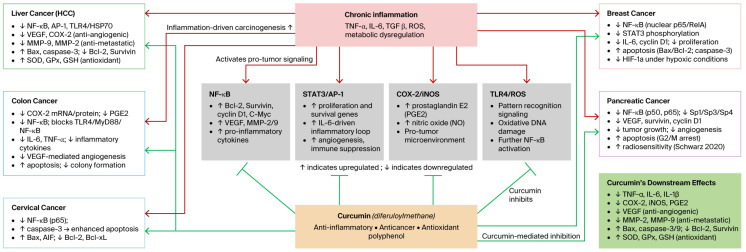
Curcumin modulates inflammation-driven carcinogenesis across multiple cancer types. Chronic inflammatory stimuli, including microbial signals, metabolic dysregulation, and environmental insults, activate key pro-inflammatory signaling pathways including NF-κB, STAT3/AP-1, COX-2/iNOS, and TLR4, leading to the expression of pro-tumor cytokines (TNF-⍺, IL-6, and IL-1β), anti-apoptotic proteins (Bcl-2 and survivin), angiogenic factors (VEGF), and invasion-related enzymes (MMP-2 and MMP-9). Curcumin inhibits these inflammatory pathways by blocking NF-κB and STAT3 activation, suppressing COX-2 and iNOS expression, attenuating TLR4/MyD88 signaling, and reducing ROS while enhancing antioxidant defenses (SOD, GPx, and GSH). In liver cancer (HCC), curcumin decreases NF-κB, AP-1, VEGF, and MMP-9 expression while inducing apoptosis via Bax/caspase-3 upregulation [89,90,91]. In breast cancer, curcumin inhibits NF-κB (nuclear p65) and STAT3 phosphorylation, decreasing IL-6-driven proliferation and promoting apoptosis [84,85,86]. In colon cancer, curcumin specifically downregulates COX-2 mRNA and protein and blocks the TLR4/MyD88/NF-κB axis, suppressing pro-inflammatory cytokines and VEGF-mediated angiogenesis [12,98]. In pancreatic cancer, curcumin suppresses NF-κB (p50/p65) and its downstream targets cyclin D1, survivin, and VEGF; induces apoptosis; and enhances radiosensitivity [92,99,100]. In cervical cancer, curcumin inhibits NF-κB activation and activates caspase-3-dependent apoptosis via the NF-κB-p53-caspase-3 pathway [94]. Green arrows (⊣) indicate inhibition by curcumin; the thin green arrow indicate the downregulation of specific molecules; red arrows indicate pro-carcinogenic activation by inflammation.

Mechanistically, curcumin has been shown to inhibit the transcriptional activity of NF-κB [77,95], a central regulator of genes involved in cell proliferation, survival, inflammation, and cell cycle progression. Figure 1 illustrates how curcumin modulates inflammation-driven carcinogenesis across multiple cancer types. At the molecular level, curcumin suppresses NF-κB signaling by inhibiting both upstream and downstream regulators. Upstream, curcumin interferes with TNF receptors, NF-κB-inducing kinases, and the IκB kinase (IKK) complex. This inhibition blocks IκBα phosphorylation and degradation, preventing p65 phosphorylation, acetylation, and nuclear translocation. Consequently, curcumin downregulates the NF-κB-dependent gene expression involved in cell proliferation (COX-2, cyclin D1, and c-Myc), anti-apoptosis (Bcl-2, Bcl-xL, and IAPs), and metastasis (VEGF, MMP-9, and ICAM-1). Overall, this leads to reduced cellular proliferation, enhanced apoptosis, and inhibition of tumor progression [38,77,96,97].

Curcumin also inhibits the JAK/STAT signaling pathway by suppressing phosphorylation of JAK1, JAK2, and STAT3, leading to downregulation of oncogenic targets such as c-Myc, MMP-9, Snail, Twist, and Ki-67. This results in reduced tumor cell proliferation, migration, and invasion; decreased tumor growth; and improved survival in glioma models [6,7,101]. Curcumin inhibits TrxR activity in HeLa cells by paradoxically shifting cells towards oxidative stress [102]. Curcumin also activates the mitogen-activated protein kinases (MAPKs) via ROS, thereby promoting apoptosis in tumor cells [103]. Curcumin also exerts immune-cell-specific effects through differential modulation of macrophages, dendritic cells, T lymphocytes, B cells, and natural killer (NK) cells [104]. For example, in normal Wistar rats, intraperitoneal injections of curcumin at 40 mg/kg/rat every 24 h for 30 days preserved Th1 cytokine production, NK-cell cytotoxicity, and macrophage activity [105]. Curcumin protects T-cell apoptosis when administered orally at 50 mg/kg, activates the JAK3–STAT5a pathway in T cells, restores BCL-2 expression in tumor-bearing mice, and prevents tumor-induced thymic atrophy through restoration of NF-κB pathway activity [106,107]. In summary, curcumin in tumor cells directly inhibits transcription factors and signaling components such as NF-κB, JAK/STAT, and ROS-generating systems by targeting NF-κB-inducing kinases, JAK/STAT kinases, TrxR, and MAPKs and induces downstream effects on genes and proteins regulated by these transcription factors via suppression of inflammatory cytokines and inflammatory enzymes (COX-2 and iNOS), as well as promotion of apoptosis via increased pro-apoptotic BAX and decreased anti-apoptotic Bcl-2 expression [103,108,109].

Despite its favorable pharmacological safety, curcumin exhibits poor oral bioavailability and limited aqueous solubility, restricting its clinical efficacy. In a phase I clinical trial, oral curcumin (8000 mg/day) was administered to 25 patients (13 men and 12 women; median age: 60 years, range: 36–77 years), yielding peak serum concentrations of 0.4–1.6 uM at 1–2 h after intake; seven patients derived clinical benefit after three months of treatment [110]. A phase II clinical trial in patients with advanced pancreatic cancer demonstrated limited but measurable biological activity of oral curcumin (8 g/day); among 21 patients, two exhibited clinical benefit, including one case of brief tumor regression and one case of disease stabilization lasting 18 months [11]. On average clinical studies reported doses ranging from approximately 2.5 g/day to 5 g/day, and phase I studies demonstrated safety up to 12 g/day despite low bioavailability [111]. The most recent clinical trials from December 2018–November 2026 with curcumin alone and curcumin in combination with chemotherapy in different cancers with completion and estimated dates of completion are listed in the review [10]. Multiple studies have shown that curcumin is present at very low concentrations in blood, tumors, and extraintestinal tissues, largely due to poor absorption, rapid metabolism, chemical instability, and swift systemic elimination. For example, oral administration of curcumin at 500 mg/kg yields a serum concentration of only 0.06 µg/mL, reflecting approximately 1% bioavailability. Its limited solubility in neutral and acidic environments, together with its propensity to undergo hydrolysis under intestinal conditions, further restricts its stability. Curcumin is rapidly metabolized in the intestine through conjugation to glucuronide and sulfate forms, with studies consistently reporting negligible levels of free curcumin and substantially higher levels of its metabolites in serum, underscoring its rapid biotransformation [112].

Curcumin also demonstrated anti-inflammatory effects in clinical studies. In a randomized placebo-controlled trial involving 80 patients with solid tumors, curcuminoid supplementation as an adjuvant therapy (180 mg/day for 8 weeks orally) significantly reduced systemic inflammatory mediators, including TNF-α, MCP-1, hs-CRP, and TGF-β, compared with the placebo [10,113]. Similarly, in a phase I trial of APG-157 (a botanical formulation containing curcumin), oral cancer patients showed reduced salivary IL-1β, IL-6, and IL-8 levels within 24 h of treatment [10,114]. Overall, clinical evidence remains limited but promising: curcumin appears safe and may provide adjunctive benefits, although it has not consistently improved survival outcomes [78,112]. Its clinical translation is hindered by poor bioavailability, variability in formulations, and a lack of large-scale trials.

In preclinical studies, curcumin has demonstrated synergistic anticancer effects when used in combination with various chemotherapeutic drugs [10]. Briefly, studies showed that the combination of curcumin with Paris saponin II induced apoptosis in lung cancer cells via PI3K/Akt pathway inhibition [115]. In breast cancer, which contains both MCF-7 and cisplatin-resistant MCF-7DDP cells, FEN1 expression was reduced in the group receiving combined curcumin and cisplatin treatment compared with cisplatin alone. Moreover, FEN1 overexpression in MCF-7 cells attenuated the chemo-sensitizing effect of 20 μmol/L curcumin on 2 μg/mL cisplatin, whereas FEN1 silencing in MCF-7DDP cells further enhanced the sensitizing effect of 20 μmol/L curcumin to 5 μg/mL cisplatin [116]. In brain cancer cells, curcumin potentiated the cytotoxicity of the EGFR inhibitors AG494 and AG1478 by causing irreversible DNA damage [117]. Curcumin has been shown to enhance the efficacy of imatinib by downregulating p210 BCR-ABL and Hsp90, and it has also been reported to potentiate the cytotoxic effects of daunorubicin [10]. Emerging evidence from colorectal cancer and gallbladder cancer mouse models suggest that inhibiting tumor-associated microbiota can be beneficial. Curcumin modulates tumor-associated microbiota by reshaping microbial composition, promoting beneficial microbial populations while suppressing tumor-promoting microbes, thereby reducing dysbiosis-associated inflammation [118,119] and, in combination with the probiotic *Lactobacillus rhamnosus* GG, reversing gemcitabine resistance in a gallbladder tumor mouse model [119]. Preclinical evidence strongly and consistently demonstrates that curcumin exhibits multi-targeted anticancer activity by inhibiting key cell-signaling pathways.

**Figure 2:**
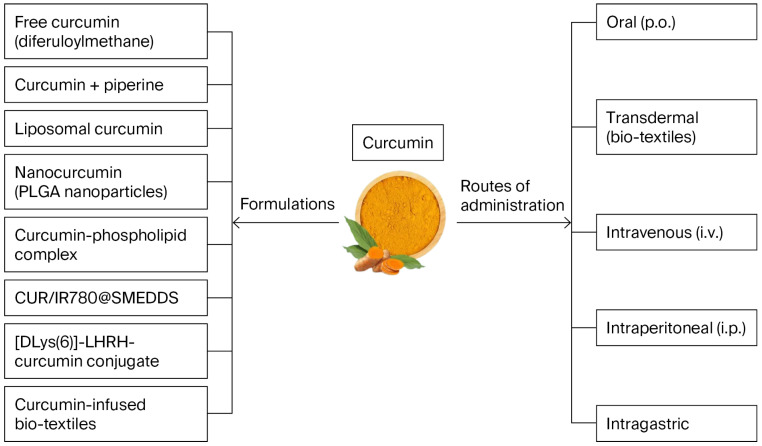
Curcumin formulations and routes of administration used in preclinical cancer studies.

Various formulations and combinations of curcumin have been tested in diverse cancer cell lines and mouse models, as summarized in Table 1 and Figure 2 [12,84,85,86,89,90,92,98,99,100,120,121,122,123]. Curcumin, administered alone at concentrations ranging from 1.3 μM to 100 μM, significantly suppressed both invasive and metastatic potential in vitro across multiple cancer cell lines, including lung cancer (6–60 μM), breast cancer (1.3–30 μM), brain cancer (25 μM), pancreatic cancer (10–100 μM), gastric cancer (4–10 μM), and leukemia (10–80 μM) [10]. Nevertheless, oral administration of curcumin enhanced the antitumor activity in the preclinical rat cancer model bearing the highly cachectic Yoshida AH-130 ascites hepatoma [124]. Strategies to improve curcumin bioavailability, such as co-administration with piperine, have shown promise in enhancing systemic absorption and therapeutic potential [125]. In rats, curcumin alone (2 g/kg) produced moderate serum levels, whereas co-administration with piperine (20 mg/kg) significantly increased serum concentrations and bioavailability by 154% by altering its pharmacokinetics. In humans, curcumin alone (2 g) resulted in very low or undetectable serum levels, whereas adding piperine (20 mg) dramatically enhanced its absorption and increased its bioavailability by 2000% without adverse effects [126].

Numerous advanced formulations of curcumin have also been evaluated. Liposomal curcumin demonstrated antitumor activity at levels comparable to or greater than free curcumin when tested at equal molar concentrations in pancreatic cancer. IC_50_ values of liposomal curcumin ranged from approximately 2.0 μM in Capan-1 cells to 2.5 μM in BxPC-3 and 37.8 μM in Capan-2 cells, whereas free curcumin exhibited IC_50_ values between 5.4 μM (BxPC-3 and Capan-1) and 46 μM (Capan-2) [99,120]. Liposomal curcumin (40 mg/kg given intravenously 3 times weekly) inhibited pancreatic carcinoma growth in murine xenograft models, suppressed tumor angiogenesis and exhibited no overt host toxicity in mice [99]. In vivo, in a pancreatic cancer mouse model where free curcumin is given orally and the a bioconjugate, luteinizing hormone releasing hormone analog-Curcumin ([DLys^6^]–LHRH–Curcumin) is given intravenously at 60 mg/kg twice weekly, the [DLys^6^]–LHRH–curcumin conjugate significantly reduces pancreatic tumor volume in a nude mouse xenograft model as compared to free curcumin. The [DLys^6^]–LHRH–Curcumin conjugate becomes a water-soluble drug without overt toxicity [100]. Liposomal curcumin and [DLys^6^]–LHRH–Curcumin improve solubility and systemic exposure, supporting curcumin’s use as an adjuvant that can be combined with standard anticancer therapies [99,100].

Nanocurcumin formulations, particularly those incorporating poly (lactic-co-glycolic acid) (PLGA), have demonstrated enhanced solubility and improved therapeutic efficacy [127,128,129,130]. PLGA-encapsulated curcumin demonstrates strong anticancer activity at substantially lower effective doses; for example, a PLGA-PAMAM-PCL nanocurcumin system reduced A549 lung cancer cell viability to about 21% after 24 h at 200 nM, with an IC_50_ observed at 50 nM and no significant toxicity detected in mesenchymal stem cells [121]. PLGA is considered safe because it degrades in vivo into glycolic acid and lactic acid, both of which are naturally metabolized through the tricarboxylic acid (TCA) cycle and ultimately excreted as water and carbon dioxide [130]. Surface modification with polymers such as polyethylene glycol helps protect PLGA nanoparticles from premature degradation by the reticuloendothelial system, thereby enhancing their stability and further improving the plasma bioavailability of curcumin following oral administration [130].

The curcumin–phospholipid complex significantly improved liver antioxidant enzyme levels and provided greater hepatoprotection than free curcumin at the same dose. These complexes also produced higher peak serum concentrations and sustained effective curcumin levels for longer durations compared to pure curcumin [131]. A hybrid self-microemulsifying drug delivery system (CUR/IR780@SMEDDS) was formulated to co-encapsulate a curcumin–phospholipid complex and the near-infrared dye IR780 and compared with suspensions of curcumin and IR780. Pharmacokinetic studies in rats demonstrated markedly improved oral absorption, with relative bioavailability increases of 743.7% for curcumin and 307.0% for IR780 compared with free compounds [122]. Free curcumin exerts antiproliferative and pro-apoptotic effects across several cancer cell lines but generally requires higher micromolar exposures in vitro, with dose–response experiments in breast cancer models showing that concentrations of 75–125 µM markedly reduce cell viability and that 75 µM functions as the IC_50_ at 24 h, leading to a sustained decrease in the mitotic index and near-complete suppression of cell division by 72 h [84]. By contrast, in metastatic 4T1 breast cancer cells, CUR/IR780@SMEDDS combined with localized NIR irradiation significantly enhanced cytotoxicity and inhibited invasion. In orthotopic 4T1 tumor-bearing nude mice, oral CUR/IR780@SMEDDS (50 mg/kg curcumin and 5 mg/kg IR780 every 2 days for 16 days) with local NIR irradiation suppressed tumor growth and lung metastasis [122]. Moreover, curcumin-infused bio-textiles, such as disposable bra inserts, have been proposed as a practical strategy for daily breast cancer prevention, delivering bioavailable curcumin transdermally via skin contact [123]. Thus, improved formulations of curcumin are emerging as powerful tools to overcome its bioavailability and stability limitations, further supporting its development as a promising chemopreventive and adjunct therapeutic agent.

In particular, oncology clinical studies have consistently demonstrated that curcumin given orally at doses up to 12 g/day is well tolerated without significant toxicity, with only mild gastrointestinal discomfort. Certain preclinical studies found potential drug interactions that warrant a more cautious and nuanced description of curcumin safety. For example, curcumin administered orally at doses of 8–12 g/day was shown to alter systemic iron metabolism in an iron-deficient mouse model that was fed a low-iron diet (5 mg iron/kg), whereas mice receiving iron-sufficient diets (12–1000 mg iron/kg) showed no changes [132]. In rabbits, pre-treatment with curcumin (60 mg/kg; orally) for three days at an interval of 24 h can enhance systemic bioavailability of norfloxacin because curcumin suppressed intestinal and hepatic UDP-glucuronyltransferase and inhibition of CYP3A4, which may delay norfloxacin’s metabolism and excretion. This may lead to a reduction in both the loading and maintenance doses of norfloxacin, suggesting that prior administration of curcumin may provide economic benefits while also minimizing drug-related side effects, as lower amounts of the drug are required [133]. Similarly, in Wistar rats, pre-administration of (100 mg/kg; orally) curcumin for 7 days affects the pharmacokinetics of warfarin and clopidogrel, with no detectable pharmacodynamic impact on anticoagulation or antiplatelet effects [134]. Some documented adverse effects of curcumin are mild and reversible, including cases of allergic contact dermatitis on skin exposure [135]. Taken together, these data support the conclusion that curcumin is generally safe and well tolerated in cancer patients at doses commonly used in clinical trials, but these findings also highlight specific contexts, such as pre-existing iron deficiency or concomitant drugs with narrow therapeutic indices, where caution and monitoring may be warranted.

## 6. Summary

Chronic inflammation is increasingly recognized as a critical enabling factor in carcinogenesis, particularly in cancer and cancer metastasis. Persistent inflammatory stimuli promote continuous recruitment of immune cells, sustained cytokine production (such as IL-6, TNF-α, TGF-β, and ROS), and activation of redox-sensitive transcription factors, thereby establishing a tumor-promoting microenvironment. Among the central mediators, NF-κB plays a pivotal role by regulating genes involved in proliferation, survival, angiogenesis, invasion, and resistance to apoptosis. Its downstream targets further amplify inflammatory signaling, enhancing tumor growth and metastatic potential. Constitutive activation of NF-κB and STAT 3 in several cancers underscores their importance as mechanistic links between inflammation and tumor progression, promoting immune evasion, enhancing survival signaling, and facilitating resistance to chemotherapy and radiotherapy.

At physiological levels, IL-6, TNF-α, TGF-β, ROS, and NF-κB function as secondary messengers that support normal proliferation and apoptotic pathways; however, their dysregulation shifts signaling toward anti-apoptotic and pro-survival programs in cancer cells, highlighting their dual roles in cancer biology. This “double-edged sword” effect provides both challenges and opportunities for therapeutic intervention, as their activity must be precisely modulated to achieve tumor-selective cytotoxicity. While chronic inflammation in the tumor microenvironment generally suppresses apoptosis and permits cancer cell survival and proliferation, curcumin can restore apoptotic signaling in inflammation-driven cancers by targeting key pathways. It inhibits the NF-κB and JAK/STAT signaling pathways, leading to downregulation of anti-apoptotic proteins (e.g., Bcl-2, Bcl-xL, and IAPs) and suppression of pro-survival gene expressions. Simultaneously, curcumin upregulates pro-apoptotic factors such as Bax and activates caspases. It also reduces inflammatory mediators (e.g., TNF-α, IL-6, COX-2, and iNOS), thereby disrupting the pro-survival tumor microenvironment and restoring apoptosis in cancer cells.

Given the central role of inflammatory pathways in tumor development, targeting these molecular circuits represents a rational therapeutic strategy. In this context, plant-derived bioactive compounds have gained significant attention. Curcumin has demonstrated the ability to suppress NF-κB and STAT 3 activation, downregulate their target proteins, modulate cytokine production, and induce apoptosis in various cancer models. Extensive preclinical research consistently shows that curcumin possesses broad anticancer properties, acting on multiple molecular targets by suppressing oncogenic signaling pathways, promoting apoptosis, and reducing inflammation and oxidative stress across diverse cancer types. In contrast, clinical evidence is still limited but encouraging curcumin appears safe and may offer benefits as an adjunct therapy, although it has not reliably improved survival outcomes. Its clinical application remains constrained by poor bioavailability, inconsistent formulations, and insufficient large-scale trials. Overall, curcumin currently appears most promising as a chemopreventive or supportive treatment rather than as primary therapy, although ongoing advances in formulation may enhance its effectiveness.

These properties position curcumin as a promising chemopreventive and adjunct therapeutic agent, yet its clinical translation continues to be limited by poor solubility, rapid metabolism, and low systemic bioavailability. In addition, combining NF-κB and STAT3 inhibitors—including curcumin and related agents—with conventional chemotherapeutics may offer synergistic benefits by targeting both tumor cells and the inflammatory microenvironment. A deeper understanding of inflammation-driven oncogenic signaling will be essential for developing more precise, mechanism-based therapeutic interventions.

## 7. Future Prospectives

The future of curcumin research is likely to be shaped by precision-medicine approaches that focus on identifying specific patient subgroups, molecular profiles, and cancer types that are most responsive. Large, well-designed randomized controlled trials will be essential to establish standardized dosing regimens, optimize formulations, and generate high-quality evidence for clinical efficacy. Given its low toxicity profile and pleiotropic effects, curcumin is an attractive candidate for combination therapy, thereby enhancing the efficacy of chemotherapy and radiotherapy, mitigating treatment-related side effects, and potentially reducing drug resistance.

Because curcumin undergoes extensive hepatic metabolism and clearance, careful monitoring of liver function enzymatic profiles will be important when developing curcumin as a drug or as an adjacent cancer treatment. The trajectory of curcumin in oncology will depend on overcoming key translational barriers. Although various formulations—such as nanoparticle-encapsulated systems, liposomal carriers, phospholipid complexes, and structural analogs—have improved curcumin’s bioavailability, further research is required to show reproducible pharmacokinetics and consistent clinical benefit. Curcumin interactions with chemotherapeutic drugs require careful evaluation in both clinical and preclinical settings. Current clinical evidence is insufficient in supporting these interactions. Therefore, further evaluation is needed.

While curcumin is unlikely to replace conventional cancer therapies, continued refinement of its formulations and targeted applications may strengthen its role as an effective adjuvant in cancer prevention and treatment.

## 8. Conclusions

Inflammation activates cellular targets that support cancer cell survival and proliferation. Collectively, the evidence highlights the intricate interplay between ROS, chronic inflammation, and cytokine signaling in shaping tumor progression, metastasis, and therapeutic responsiveness. Curcumin’s spectrum of pharmacological activities, including anti-inflammatory and antioxidant properties, as well as its subsequent chemopreventive and chemotherapeutic uses, allows it to be developed as a cancer adjuvant therapeutic drug. Curcumin remains a promising candidate for development as an adjuvant therapeutic agent in cancer, particularly in inflammation-driven malignancies.

## Figures and Tables

**Table 1:** Curcumin formulations tested on various cancer cells.

Curcumin Formulation	Cancer Cell Line(s)	Study (Year)	Key Findings
Free curcumin	SK-Hep-1 and Huh-7 (liver)	Lin et al., 1998 [89]	Inhibited invasion and migration of HCC cells via MMP-9 suppression
Free curcumin	HepG2 (liver)	Cao et al., 2007 [90]	Induced apoptosis through a mitochondrial pathway; promoted cytochrome c release
Free curcumin	MCF-7 and MDA-MB-231 (breast)	Goksu Guneydas and Topcul, 2022 [84]	Dose-dependent anti-proliferative effects in luminal A and triple-negative breast cancer cells
Free curcumin	Panc-1 and MIAPaCa-2 (pancreatic)	Schwarz et al., 2020 [92]	Radiosensitized pancreatic cancer cells; enhanced radiation-induced apoptosis and G2/M arrest
Curcumin + piperine (nanoparticles)	HCT-116 (colon)	Puri and Arora, 2025 [12]	Synergistic anticancer effect; enhanced cellular uptake; IC_50_ lower than free curcumin
Liposomal curcumin	Pancreatic carcinoma (in vivo)	Li et al., 2005 [99]	Activity comparable to or greater than free curcumin; inhibited tumor growth and suppressed angiogenesis in murine xenografts
Liposomal curcumin	AsPC-1 and BxPC-3 (pancreatic)	Mahmud et al., 2016 [120]	Potent anticancer activity on pancreatic adenocarcinoma cells; less toxic to normal cells than free curcumin
Nanocurcumin (PLGA)	A549 (lung)	Almajidi et al., 2024 [121]	IC_50_ of 50 nM at 24 h; 6–8-fold upregulation of apoptotic genes (caspase-9 and Bax); no toxicity in normal cells
Nanocurcumin (PLGA)	HCT-116 (colon)	Waghela et al., 2015 [98]	Inhibited colony formation and cell migration; enhanced anti-cancer activity vs. free curcumin
Nanocurcumin (PLGA)	MDA-MB-231 (breast) and A549 (lung)	Khan et al., 2018 [85]	Reduced HIF-1a and nuclear p65 expression; enhanced anti-cancer activity under hypoxic conditions
Curcumin-phospholipid complex	HC11 and BME-UV (mammary epithelial)	Cucuzza et al., 2008 [86]	Induced apoptosis in mammary epithelial cells via the STAT-3 signaling pathway
CUR/IR780@SMEDDS	4T1 (breast and metastatic)	Liu et al., 2019 [122]	Enhanced oral bioavailability; inhibited lung metastasis of breast cancer in vivo
[DLys^6^]-LHRH-Curcumin	MIAPaCa-2, Panc-1, and BxPC-3 (pancreatic)	Aggarwal et al., 2011 [100]	Water-soluble conjugate selectively targeted and inhibited pancreatic cancer cell growth; enabled i.v. administration
Curcumin-infused bio-textiles	Transdermal delivery (proof of concept)	Atlan et al., 2019 [123]	Delivered targeted transdermal curcumin therapy through simple skin contact

Note: Nanocurcumin is PLGA-encapsulated curcumin, PLGA, poly (lactic-co-glycolic acid); CUR/IR780@SMEDDS, Hybrid self microemulsifying curcumin-phospholipid complex-near-infrared dye; [DLys^6^]-LHRH-Curcumin (a bioconjugate, luteinizing hormone releasing hormone analog-Curcumin; i.v. intravenous.

## Data Availability

No new data were created or analyzed in this study. Data sharing is not applicable to this article.

## Abbreviations

The following abbreviations are used in this manuscript:

IL-6	Interleukin-6
TNF-α	Tumor necrosis factor-α
TGF-β	Transforming growth factor-β
TME	Tumor microenvironment
NF-κB	Nuclear factor kappa B
COX-2	Cyclooxygenase-2
iNOS	Inducible nitric oxide synthase
STAT3	Signal transducer and activator of transcription 3
ROS	Reactive oxygen species
TRADD	TNFR-associated death domain
TNFR	Tumor necrosis factor (TNF) receptor
TRAF	TNF receptor-associated factor
OSCC	Oral squamous cell carcinoma
PLGA	poly(lactic-co-glycolic acid)
